# Fluid Attention in Education: Conceptual and Neurobiological Framework

**DOI:** 10.3389/fpsyg.2021.704443

**Published:** 2021-09-28

**Authors:** Brian Bruya, Yi-Yuan Tang

**Affiliations:** ^1^Department of History and Philosophy, Eastern Michigan University, Ypsilanti, MI, United States; ^2^Department of Psychological Sciences, Texas Tech University, Lubbock, TX, United States

**Keywords:** voluntary attention, involuntary attention, fluid attention, postvoluntary attention, effortless attention, effortful attention, ADHD, intrinsic motivation

## Abstract

Attention is indispensable to our learning, performance, relationships, health, and daily life, and yet laboratory studies of attention have only scratched the surface of these lived varieties of attention. In this article, we begin with William James' theory of derived involuntary attention, which has largely been ignored in laboratory research. We then show that there is a gap in our attention vocabulary and the theory that underpins it, which depend on an incomplete voluntary/involuntary dichotomy. The negative effects of this dichotomy stretch beyond laboratory research to clinical diagnosis, influencing how we understand so-called attention deficits. To fill the gap between voluntary and involuntary, we introduce a third kind of attention—fluid attention (also called postvoluntary attention), which is goal-directed and selective, like voluntary attention, but also effortless and drawn to its source, like involuntary attention. Fluid attention is a rediscovery of James' derived involuntary attention. A distinguishing feature of fluid attention is its motivational component, which, we show, neurophysiologically also reveals a gap in the neurocognitive literature on attention. Recognizing fluid attention as fundamentally motivational allows ADHD to be redefined as a motivational rather than an attentional deficit, which we go on to show has significant implications for both special and regular education.

The stream of our thought is like a river. On the whole easy simple flowing predominates in it, the drift of things is with the pull of gravity, and effortless attention is the rule.

-William James, *Principles of Psychology*

## Introduction

This article is a first attempt at introducing the notion of *postvoluntary attention* into the English-language cognitive science literature. The term originates in the Russian-language work of Nikolaj Dobrynin, only one article of which has been translated into English to date (Dobrynin, 1968). Our aim is not just to explain Dobrynin's idea but to adopt it, showing how there is a gap in current theory without it and how adopting the idea in future research programs can improve both theory and concrete applications. We also introduce the synonym: *fluid attention* as a more descriptive alternative.

We'll begin with the notion of active learning to motivate this project and return to it at the end of the article as an example of how the idea of postvoluntary attention can be effectively applied. Of countless education fads that have come and gone, one idea that has had lasting power has been that of active learning. Early in the twentieth century, something like it was promoted by such educational luminaries as Maria Montessori (Montessori and Holmes, 1912) and Dewey (1916), but the label “active learning” itself was introduced only in the 1980s, when a variety of research programs centered on it (Bonwell and Eison, 1991). It is now the gold standard at all levels of education (Prince, 2004; Freeman et al., 2014).

Active learning, according to Bonwell and Eison (1991) is marked by the following characteristics (a list which is consistent with other accounts):

- Students are involved in more than listening
- Less emphasis is placed on transmitting information and more on developing students' skills
- Students are involved in higher-order thinking (analysis, synthesis, evaluation)
- Students are engaged in activities (e.g., reading, discussing, writing)
- Greater emphasis is placed on students' exploration of their own attitudes and values

Benware and Deci (1984) reduced active learning to its most minimal form by providing two sets of students the same material to be learned with the only difference being what to expect once the material was learned—the method of learning was left up to the subjects. In this way, Benware and Deci demonstrated that active learning is not about activities, *per se*, but about intrinsic vs. extrinsic motivation. As the final bulleted item above also suggests, active learning mobilizes students' intrinsic motivation.

Similarly, there is increasing evidence that Attention-Deficit/Hyperactivity Disorder (ADHD) is less about an attention deficit, *per se*, and more about a motivation deficit, primarily intrinsic motivation (McInerney and Kerns, 2003; Volkow et al., 2011; Morsink et al., 2017). Thus, in both regular education and special education, learning depends importantly on engendering intrinsic motivation in students. To the extent that learning also requires students to pay attention, a crucial question arises: what is the relationship between intrinsic motivation and attention? The current paradigm of attention, due to the nature of the paradigm itself, overlooks this question, and it is our aim in this article to unite attention and intrinsic motivation under the rubric of fluid attention.

### Voluntary and Involuntary Attention

There is a distinction between voluntary and involuntary attention (Anderson et al., 2011) that goes back to the time of James (1908). Currently, the preferable language is often “endogenous” and “exogenous,” as in the following passage from a recent psychology textbook, “In endogenous attention we voluntarily select objects to attend to…. Exogenous attention is the involuntary capture of attention by stimuli” (Watson and Breedlove, 2005). Sometimes, the focus is on temporality, and the terminology “sustained” and “transient” is used (Liu et al., 2005). Sometimes, directional terminology of “top-down” and “bottom-up” is preferred (Bowling et al., 2020). Sometimes, the teleological “goal-directed” vs. “stimulus-driven” is preferred (Corbetta and Shulman, 2002)^1^. Regardless of the vocabulary used, voluntary attention is the process of intentionally orienting, or directing, one's attention to a perceptual (outside the mind) or conceptual (inside the mind) source. Psychologists often refer to this voluntary process as “selective attention.” Involuntary attention is drawn reflexively to a perceptual source.

James conceived of attention less in terms of laboratory tasks and more in terms of everyday life. As such, he discussed another dimension of attention, using the vocabulary of “immediate” and “derived.” *Immediate attention* is attention that is directly relevant to the person in a way that James specifically refers to as “interesting” (p. 416). *Derived* (or apperceptive) attention is relevant only indirectly, through layers of association, and is goal-relevant. *Voluntary* attention, he says, is experienced as effortful, and *involuntary* attention is experienced as effortless.

Both derived and immediate attention for James can be involuntary (see Table 1). The kind of attention that most children deploy most of the time, he says, is *immediate involuntary attention*, jumping from one salient object of attention to another: “strange things, moving things, wild animals, bright things, pretty things, metallic things, words, blows, blood, etc., etc., etc.” (p. 417). Adults, however, who are able to make attenuated intellectual associations more readily can become absorbed in reveries for long periods of time, effortlessly blocking out distractions, even shielding themselves from pain. Geniuses, James says, due to their innate originality, are particularly inclined to this state of absorption, which he refers to as *derived involuntary attention* (also absent-mindedness).

**Table 1:**
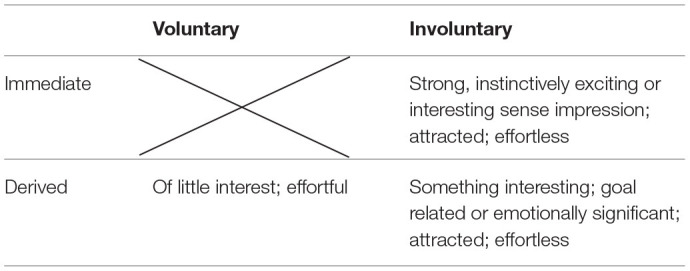
Three kinds of attention according to William James.

Unlike geniuses, most adults, James says, need to invest effort in order to maintain attention, so that they can accomplish important tasks. In fact, he says, it is an essential skill that needs to be cultivated. This is *derived voluntary attention*. It is, he says, “the very root of judgement, character, and will” (p. 424). It is very difficult, however, according to James, to sustain attention on an uninteresting subject and requires repeated efforts of renewing flagging attention.

James ends his discussion of the varieties of attention on the topic of education:

The only general pedagogic maxim bearing on attention is that the more interest the child has in advance in the subject, the better he will attend. Induct him therefore in such a way as to knit each new thing on to some acquisition already there; and if possible awaken curiosity, so that the new thing shall seem to come as an answer, or part of an answer, to a question pre-existing in his mind (p. 424).

It is important to note that James spends most of his discussion of the varieties of attention on the two kinds of involuntary attention, whereas recent scientists have done most of their research on the one kind of voluntary attention. Evidence suggests that a person is in a mind-wandering state nearly half of the time (Killingsworth and Gilbert, 2010). The narrow focus by scientists on voluntary attention has been so extreme that it has even led some scientists to conceive of attention as only effortful—effortless attention being, according to them, theoretically impossible (Bruya and Tang, 2018)—or as only selective (Yantis, 2002). Below, we show why James' view should be pursued and his so-called derived involuntary attention should be a focus of more research (but substituting the terms “postvoluntary attention” or “fluid attention” and defining it with more precision).

### Ambiguity in the Voluntary/Involuntary Distinction. What Do You Call Attention That Is Neither Voluntary Nor Involuntary?

In the common voluntary/involuntary distinction, voluntary attention is goal-directed and effortful, while involuntary attention is attracted to (captured by, drawn to) its source and effortless. But what about attention that is goal-directed, attracted to its source, and effortless, as in James' derived involuntary attention? James says, “the topic once brought back, if a congenial one, develops; and if its development is interesting, it engages the attention [involuntarily] for a time” (p. 420). The suggestion, here, is that there can be a transition from voluntary attention to involuntary attention. Because involuntary attention is commonly categorized as reflexive by the current paradigm, there is no room in the category for the notion of interest. In a very real sense, current attention theory remains strongly wedded to a primitive behaviorism, in which non-voluntary attention is a matter only of simple stimulus and response.

Consider the Stroop task. The dominant response, which must be effortfully overridden, is to read the word in the task rather than identify its color. There have been countless studies on both the dominant and non-dominant responses in the Stroop task, and when it comes to categorizing kinds of attention and conceptualizing an overall attention paradigm, the non-dominant response clearly belongs to the category of voluntary, or selective, attention. But what about the dominant response? It doesn't quite fit the category of voluntary attention because it is not effortfully selected. And it doesn't quite fit the category of involuntary attention because involuntary attention is generally understood as being attracted to a different task or activity than the one currently under attention. Reading in the Stroop task is an involuntary selection in a selective attention task, in which a subject is instructed to attend to the non-dominant response. This kind of non-intentional attending seems distinct from involuntarily attending to the sound of a door slamming shut.

There is also the case of attention that is sustained (ordinarily equated with voluntary) but experienced as effortless, as in reading an absorbing novel. By standard definitions, such an activity is neither voluntary nor involuntary. And when the mind wanders away from the novel toward some remotely goal-relevant concern, that kind of attention also seems neither voluntary nor involuntary by standard definitions.

Although the voluntary/involuntary distinction is regularly used in attention research, there remain gaps in its applicability to real-world events and activities. Below, we attempt to, at least partially, fill this gap with the notion of fluid attention, elaborating its applicability to real-world events and activities. We take ADHD as an example to further elaborate the concept of fluid attention.

### What Does “AD” Stand for in “ADHD”?

According to the *Diagnostic and Statistical Manual of Mental Disorder* (Reynolds and Kamphaus, 2013), attention deficit occurs when:

Six (or more) of the following symptoms have persisted for at least 6 months to a degree that is inconsistent with developmental level and that negatively impacts directly on social and academic/occupational activities:
  - Often fails to give close attention to details or makes careless mistakes in schoolwork, at work, or during other activities (e.g., overlooks or misses details, work is inaccurate).
  - Often has difficulty sustaining attention in tasks or play activities (e.g., has difficulty remaining focused during lectures, conversations, or lengthy reading).
  - Often does not seem to listen when spoken to directly (e.g., mind seems elsewhere, even in the absence of any obvious distraction).
  - Often does not follow through on instructions and fails to finish schoolwork, chores, or duties in the workplace (e.g., starts tasks but quickly loses focus and is easily sidetracked).
  - Often has difficulty organizing tasks and activities (e.g., difficulty managing sequential tasks; difficulty keeping materials and belongings in order; messy, disorganized work; has poor time management; fails to meet deadlines).
  - Often avoids, dislikes, or is reluctant to engage in tasks that require sustained mental effort (e.g., schoolwork or homework; for older adolescents and adults, preparing reports, completing forms, reviewing lengthy papers).
  - Often loses things necessary for tasks or activities (e.g., school materials, pencils, books, tools, wallets, keys, paperwork, eyeglasses, mobile telephones).
  - Is often easily distracted by extraneous stimuli (for older adolescents and adults, may include unrelated thoughts).
  - Is often forgetful in daily activities (e.g., doing chores, running errands; for older adolescents and adults, returning calls, paying bills, keeping appointments).

Items (a) through (i) refer to deficits in voluntary, selective attention, reflecting the tendency among scientists to define attention narrowly as voluntary, or selective.

It has been shown in prior literature that ADHD, rather than describing deficits of attention, *per se*, may describe deficits of motivation. For example, using PET scans, Volkow et al. (2011) demonstrated “decreased function in the brain dopamine reward pathway in adults with ADHD” (p. 1,147; see also Ellison-Wright et al., 2008; Nakao et al., 2011). According to Volkow and colleagues:

These findings are consistent with the clinical recognition that attentional deficits in individuals with ADHD are most evident in tasks that are boring, repetitive and considered uninteresting (that is, tasks or assignments for which intrinsic motivation is low). However, the correlational approach in our study does not allow us to assess which of these dimensions is more primary; the motivation deficit produces inattention as opposed to the attention deficit resulting in decreased motivation (p. 1,151).

This question has been answered by several studies on video game playing in children diagnosed with ADHD. In these studies, children who scored poorly in laboratory tests of attention performed as well as control subjects in video games that demand the same kind of attentional abilities as the laboratory tests (Lawrence et al., 2002; Shaw et al., 2005; Bioulac et al., 2014). What seems to be obvious from these studies is that while subjects (who have been diagnosed with ADHD) have a difficult time paying attention to laboratory tasks and, as Volkow and colleagues say, boring, repetitive, and uninteresting tasks, they have no trouble paying attention to something that they find interesting and intrinsically rewarding.

The very notion of ADHD, itself, then, along with the implications that the prevalent diagnosis for school-aged children has on educational methods, seems in need of revisiting. Short of turning our educational systems into extended video games, what can be done about harnessing children's innate attentional abilities? Perhaps the first step is to admit a third kind of attention into our attentional paradigm.

### Postvoluntary Attention, a Definition

Under standard definitions, the kind of attention that children are engaged in while playing video games is not voluntary attention (because it is not effortful), nor is it involuntary attention (because it is goal-directed). According to N. F. Dobrynin, a Soviet psychologist who was active from the 1920s to 1950s, the missing ingredients in laboratory attention studies are the personality and the activity, ideas traceable to Soviet psychologists L. Vygotsky and A. N. Leontiev (Dormashev, 2010). According to Dobrynin (1968):

The selectivity of psychic activity is explained by the total development of the personality in a definite social context. The individual depends on this context. The psychic activity of an individual is directed to that which has the greatest importance for him at the given moment. Attention is *the direction and concentration* of the psychic activity and the preservation of this selected activity, and concentration means absorption in the given activity and distraction from everything else. If direction and concentration are involuntary then one speaks of “involuntary attention.” If they are tied in with a consciously set purpose, one speaks of “deliberate” attention. Side by side with these two basic types of attention, N. F. Dobrynin proposed distinguishing a third, and from his point of view a very important form of attention, which he called “postvoluntary” attention. This involves those cases when there is a conscious, premeditated accomplishment of activity connected with the absorption of a person by the given activity and not requiring volitional efforts (p. 275–276).

The model of attention offered by Dobrynin resembles James' in its insistence on real-world conditions and the importance of the subjectivity of the individual. Rather than an alienated stimulus of no relevance to the person, the topic of attention for Dobrynin and James is something of relevance to the person, who has a network of internal preferences that influence attention. However, according to James, “There is no such thing as voluntary attention sustained for more than a few seconds at a time. What is called sustained voluntary attention is a repetition of successive efforts which bring back the topic to the mind” (420). As if responding to James, Dobrynin says:

In the psychological literature the constant fluctuations of attention are always stressed, with the fact being pointed out that with every strong concentration, attention can only last for 1.5-2 seconds, after which it weakens and only then restores itself again.… Attention can, however, be maintained on a definite channel of activity if this activity is prolonged (p. 277).

Referring to his own studies, Dobrynin continues:

Thus, the original hypothesis to the effect that prolonged attention (macroattention) consisted of short periods of intense attention (microattention) alternating with short periods of weakened attention, was not confirmed. The subject could work for 20 minutes without being distracted for even one third of a second (p. 278).

Dobrynin then describes his work with children in the classroom:

The assumption emerged that longer periods of intense attention existed alternating with periods of weakening of attention. Observations seemed to show that during assignments, every few minutes after intense work some type of movement occurs, some sort of noise, as it were some sort of weakening of attention. With the purpose of studying this problem, pupils in the 2-5^th^ grades were given the task of copying a familiar text for 40 minutes. Every 30 seconds a signal was given, and the participants placed the proper numbers under the letters which they had just written.
The analysis of most of the tests indicated that there was no alternation of intense or weakened attention, at least no periodic alternations of the two were detected. Pupils between 12-14 years of age would work continuously for 40 minutes without interruption (p. 278).

Dobrynin explains this result as follows:

In the experiments mentioned, [attention] was elicited by continuous signals (every 30 seconds) forcing the students not to weaken attention. It is also well known that interesting gripping work can continue for hours without any sort of intermissions. Consequently, length or stability is not connected with any compulsory rhythm. Attention can be maintained continuously without distraction even for one third of a second depending on its being sustained by continuous efforts of will or on the fascination of the activity itself. It is important, however, that this activity be accomplished actively. The task of holding attention consists of properly organizing this activity (p. 278).

Above, we saw that James would in some sense agree. Recall that James said, “the topic once brought back, if a congenial one, develops; and if its development is interesting, it engages the attention [involuntarily] for a time” (p. 420). The difference of opinion between James and Dobrynin comes down to one of nomenclature. James says that voluntary attention cannot be sustained, while involuntary attention can be. Dobrynin says merely that attention can be sustained, but through his examples, we can see that he is also talking about a transition from what James would call voluntary to involuntary attention.

Dobrynin takes us a step forward because James' nomenclature is in conflict with current nomenclature. In current nomenclature, sustained attention refers only to selective voluntary attention. Involuntary attention is, by definition, transient. Dobrynin offers us a third kind of attention that is effortlessly sustained within an activity that the subject finds interesting.

From the above, we can postulate three distinct kinds of attention:

*Voluntary attention*: attention that is selective, effortfully sustained, and goal-directed.

*Involuntary attention*: attention that is transient and effortlessly drawn to its object.

**Figure 1:**
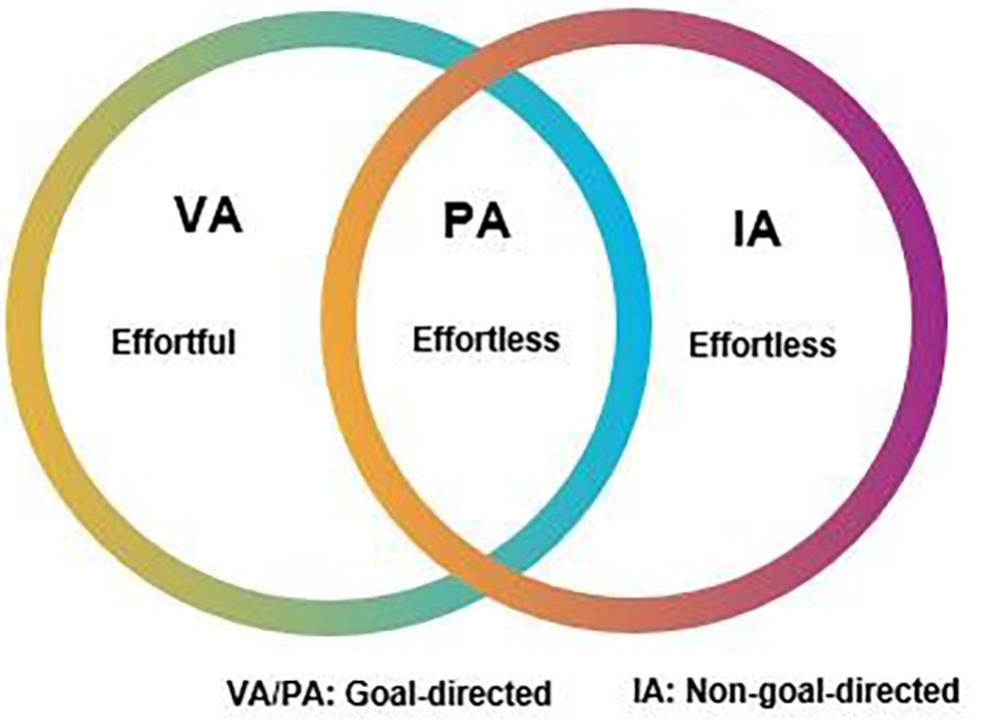
Voluntary attention (VA), postvoluntary attention (PA), and involuntary attention (IA).

*Postvoluntary attention (fluid attention)*: attention that is selective, goal-directed, drawn to its object, and effortlessly maintained, see Figure 1.

Dobrynin notes that the reason he calls it “postvoluntary” attention is that before one has mastered an activity, it takes voluntary attention to learn the required rules and skills. Over time, there is a transition to postvoluntary attention that is self-sustaining. We propose “fluid attention” as a synonym for “postvoluntary attention” because it is descriptive of the subjective, effortless experience.

We mentioned above that in the psychology and cognitive science literature, involuntary attention is often viewed in one-dimensional, behavioristic terms of stimulus and response. In this paradigm, the salience of stimuli plays a key role. The notion of fluid attention allows us to broaden our notion of salience from a stimulus-response model to a sensitivity-and-responsiveness model (Bruya, 2010). Salience is generally understood as depending on perceptual features of the object—to which all subjects will react in more or less the same way to the same salient stimulus. Scientists studying attention in the laboratory often avoid using familiar activities or allowing subjects to habituate to the activity, because then attentional set must be accounted for. Attentional set occurs when a subject is ready to differentially respond to certain stimuli or has developed differential internal responsiveness. We may call the biasing, or priming, of attentional set *learned salience* (Ghazizadeh et al., 2016), which is in contradistinction to *innate salience* (often described as stimulus-driven salience), as described just above.

### Attention and Motivation

When psychologists study attentional set, it is studied in the laboratory using simple tasks in which subjects are instructed to attend to objects under cued conditions. The first step in identifying the brain region related to attentional set was differentiating monitoring from control (MacDonald, 2000). Corbetta and Shulman (2002) identified the main neural systems that are recruited in voluntary and involuntary attention. Voluntary attention is controlled in a top-down fashion [with some bottom-up input (i.e., attentional set)] by the dorsal frontoparietal network [anterior intraparietal sulcus (IPS), superior parietal lobule (SPL), post-central sulcus, and the intersection of the precentral and superior frontal sulci]. Involuntary attention is controlled in a bottom-up fashion by the right ventral frontoparietal network [temporoparietal junction (TPJ) and ventral frontal cortex (VFC)].

Dosenbach et al. (2008) updated the Corbetta and Shulman model, separating off attentional set, or set-maintenance, from top-down control. They say that the frontoparietal network [dorsolateral prefrontal cortex (DLPFC), inferior parietal lobule (IPL), dorsal frontal cortex (DFC), IPS, precuneus, and middle cingulate cortex (MCC)] is recruited for adaptive control—that is, top-down responses that “initiate attentional control in response to cues… and process performance feedback on a trial-by-trial basis to adjust control settings” (p. 102). Distinct from that is the cingulo-opercular network [anterior prefrontal cortex (APFC), anterior insula / frontal operculum (AI/FO), dorsal anterior cingulate cortex / medial superior frontal cortex (DACC/MSFC), and thalamus], which “carries set-maintenance activity that spans the entire task epoch” (p. 102). Findings regarding set-maintenance often rely on a cue-delay-target task, in which a target is first cued, and then the subjects (humans or monkeys) must wait, maintaining the relevance of that cue until the target appears.

The tasks referred to by Dosenbach and colleagues are vigilance tasks, requiring little effort. The subject waits for an expected stimulus. By comparison, the tasks in Corbetta and Schulman are working memory tasks and comparatively effortful. This difference in task may very well explain why Corbetta and Shulman find set maintenance in the dorsal attention network and Dosenbach and colleagues find it ventral areas. In fact, it suggests that set maintenance is itself dynamic and occurs in both effortful (top-down) and effortless (bottom-up) tasks.

A common test of voluntary attention, in both experimental cognitive psychology and increasingly in psychotherapy, is the Attention Network Test (ANT), which tests for three purportedly distinct attention networks—alerting, orienting, and executive control. Alerting is the kind of vigilance in the studies above (Dosenbach et al., 2008). It can be thought of as a pre-condition of focused attention, a state in which the person is receptive to input. Orienting and executive control fall under the category of voluntary attention, as the first stage and then as sustaining stages. Executive attention as measured in the ANT involves activation of the anterior cingulate cortex and the dorsolateral prefrontal cortex (Fan et al., 2005).

More recently, several researchers have studied the neurobiology of intrinsic motivation, with promising results. A major rubric under which intrinsic motivation is studied is the topic of curiosity. Kang et al. (2009) defined “curiosity” as anticipation of rewarding information, and using trivia and word matching tasks, found activation of curiosity in the caudate nucleus (dorsal striatum), prefrontal cortex, and hippocampus. Similarly, using a stopwatch task, Murayama et al. found activation of curiosity in the caudate and left prefrontal cortex (Murayama et al., 2010). These studies suggest that learning is perceived by subjects as rewarding in and of itself, without the need for external reward.

The above studies are supported by several other studies that simultaneously complicate both the construct of intrinsic motivation and the question of its neural correlates. Using trivia tasks, correlates of curiosity in the ventral rather than the dorsal striatum were detected (Gruber et al., 2014; Ligneul et al., 2018), but these have been attributed to curiosity relief rather than curiosity itself (Ligneul et al., 2018). Jepma et al. (2012), using a task involving blurry pictures that were subsequently resolved, found activation across the basal ganglia and in the insula and orbitofrontal cortex, as well. Complicating matters even further, van Lieshout et al. (2018), using a precisely calibrated virtual task involving marbles in a vase, attributed the activation of curiosity induction to the inferior parietal lobule, curiosity relief to the insula, and found no relevant activation in the striatum. Consistent with van Lieshout et al. (2018), Lee and Reeve (2017), using a trivia task, found activations of intrinsic motivation in the insula, as did Lee et al. (2012), using an intrinsic motivation phrasing task. Contrary to all of the above, Marsden et al. (2015), using word puzzles, found that intrinsic motivation is negatively correlated with activation in the caudate, insula, and hippocampus; and Mizuno et al. (2008), under the rubric of academic motivation and using an n-back task, found activation in the putamen but not in other parts of the basal ganglia.

Although the above studies are often in conflict and use distinct or overlapping methods and conceptual constructs, they largely point in the direction of the striatum and/or the insula playing an important role in intrinsic motivation^2^. According to Tricomi and Fiez (2008), caudate activation reflects task success and may also reflect the solidification of knowledge and a sense of agency. Consistent with Tricomi and Fiez (2008); Leong et al. (2017), using a cleverly calibrated picture task to measure reinforcement learning, concluded that activity in the striatum is associated with prediction error. Kaplan and Oudeyer (2007) propose a theory in which dopamine signals in the basal ganglia reflect “progress niches,” where prediction error is expected to decrease, and so is a consequence, not a cause, of learning. Moving in a similar direction but with somewhat different terminology, Berridge (2012) proposes that dopamine surges reflect neither pleasure nor knowledge but motivation—that is, wanting, rather than liking or learning.

We propose that using the widely used ANT to test for attention deficits misses the crucial aspect of intrinsic motivation. When a child or adult is engaged in an absorbing task, there very likely will be activation in the mesolimbic dopamine network (Naqvi and Bechara, 2009; Schweinhardt et al., 2009) that will be absent in ANT tasks. Thus, to the extent that ADHD is a deficit of motivation (Zentall and Lee, 2012; Smith et al., 2020), the ANT will miss crucial aspects of it.

We propose that unlike voluntary and involuntary attention, fluid attention activates the mesolimbic dopamine system (MDS). MDS does not activate only for intrinsic motivation, as it also reflects extrinsic motivation, which we will address directly in the following section^3^.

## Discussion

### Fluid Attention in Education, a Roadmap for the Future

Despite decades of scientific study of the topic of attention and many advances in understanding the neural basis of attention (Posner and Rothbart, 2007; see also Petersen and Posner, 2012 for a review), there is still much that is uncertain. Above, we present a picture of unified progress in the neurobiology of attention research, but this glosses over fundamental disagreements and dissenting voices. Across the field, there is little agreement about the specific neuroanatomical components of the major streams of attention, even of the number of streams. There is even less agreement about how functional networks (alerting, orienting, executive) map onto to the neuroanatomical (dorsal, ventral) streams. In a recent paper, Hommel et al. (2019) review the lack of progress in settling specific debates related to the neuroanatomy of attention, finally proposing that we drop the term “attention” altogether.

Although there is little unity in the specifics of attention research, there is general unity that there is a top-down aspect of attention anchored in a frontoparietal network (DFC, superior parietal lobe, frontal eye field, and intraparietal sulcus) and a bottom-up aspect of attention anchored in a more ventral parietal network (intraparietal lobe, inferior frontal gyrus, middle frontal gyrus, tempo-parietal junction), both or either of which also implicate the anterior insula / frontal operculum in certain tasks. As explained above, none of these anatomical sites necessarily implicate the MDS, where we propose fluid attention may be anchored. Let us go a step further, though space prevents us from elaborating, and propose two further potential anatomical anchors of fluid attention.

First is Broca's area, known primarily for anchoring linguistic comprehension and expression. A key element of language attributed to Broca's area is syntax (Koelsch et al., 2005; Progovac et al., 2018), including that of music listening (Patel, 2003). Because activities that invite attentive absorption involve a significant mastery of syntax (Bruya, 2010, in press), activation in Broca's area should figure prominently. This is distinct from stimuli and tasks involving involuntary and voluntary attention that are typically sparse in constitutive syntax.

Second, reward and a sense of agency in the MDS also implicate the ventromedial prefrontal cortex (VMPFC) when they involve social connection (Eisenberger and Cole, 2012; Roy et al., 2012; Redcay and Schilbach, 2019). Given the fundamentally social nature of education, games, play, etc., we propose that in addition to MDS, the VMPFC, including the adjacent anterior cingulate cortex, will likely also be active in many activities where fluid attention is achieved.

Testing these two anchors of fluid attention will be challenging for the reason that the activities that elicit fluid attention are not amenable to easy laboratory study (Moller et al., 2010). One potential way forward is studying simple computer games in an fMRI machine, games that are absorbing and enjoyable to the participant, potentially activating their motivational centers and syntax following.

Following from our discussion in Attention and Motivation section above, we propose that the main deficit in ADHD is the student's inability to take ownership of the learning process. An admittedly boring task is perceived by the student as alienating, meaningless, or insignificant. The responsibility of the educator—and this is true for all students but especially so for ADHD students—is to make the material meaningful for the student. This perspective is consistent with Dobrynin's own writing, in which he says that postvoluntary attention is a matter of absorption in an activity, which in the case of education involves the student's interest and often the caring guidance of an instructor. He says:

Pupils will work with interest on something which, though it may be difficult is yet susceptible of accomplishment, which can give them a sense of some achievement, something at which they receive encouragement and support from a teacher who is sufficiently demanding, but at the same time tactful and considerate enough toward the pupils (Dobrynin, 1968, p. 282).

Dobrynin speaks of students generally, but what of students with learning disabilities?

An example of instructor involvement inducing learning improvement in students with learning disability can be found in the work of Zentall and Lee (2012), who introduced an intervention for students with reading disability. Primary school students diagnosed with a reading disability were given individualized attention involving three kinds of intervention: (1) positive feedback about prior performance relative to mastery (intrinsic) goals, (2) positive labeling, and (3) encouragement toward performance (extrinsic) goals. Students with reading disability improved their scores in both reading fluency and reading comprehension post-intervention, compared to control students, who were given brief instructions only. Interestingly, it was not only the reading disability students who improved. Students with grade-level or above reading ability improved by about the same amount, compared to controls.

When fluid attention is explained in terms of games and play and then it is proposed that fluid attention be applied to education, it is easy to assume that the overall proposal is simply to gamify education. That can be one solution, but we think it is better to think in terms of active learning. The important transition that needs to be made is one from insignificance or meaninglessness to significance or meaningfulness. This can be done by making material fun, of course, but it can also be done through encouragement and human connection, which in the end may be easier to become self-sustaining as the student matures.

The term “postvoluntary” is an apt description of the phenomenon under discussion here, as it is cultivated via, and follows, voluntary attention. It is not, however, descriptive of the attentional process itself. Because postvoluntary attention is experienced as effortless in activities that often involve a combination of vigilance and rapid but fluid response to unpredictable cues, we propose the use of the more descriptive term “fluid attention” as a synonym for “postvoluntary attention.”

To summarize, we propose that the current paradigm of voluntary and involuntary attention be revised to include a third kind of attention—postvoluntary, or fluid, attention. This notion fills a gap in research on attention that has traditionally focused on prompted, rigid, stipulated laboratory tasks. A side-effect has been the adoption of this paradigm into educational psychology, in which students who have trouble taking ownership of extrinsically motivated, boring tasks are diagnosed with a medical cognitive disability.

Over a century of research on active learning, by contrast, has taught us that learning is best achieved when it engages a student's intrinsic motivation and is conducted by an encouraging teacher, thus engaging the motivational and social brain networks. We propose that the introduction and use of the terms “postvoluntary attention” and “fluid attention” will facilitate more successful research and application of the inherent connection between attention and education.

## Data Availability Statement

The original contributions generated for the study are included in the article. Further inquiries can be directed to the corresponding author.

## Author Contributions

BB and Y-YT conceptualized the outline of the manuscript together and contributed to manuscript revision, read, and approved the submitted version. BB wrote the first draft. Both authors contributed to the article and approved the submitted version.

## Conflict of Interest

The authors declare that the research was conducted in the absence of any commercial or financial relationships that could be construed as a potential conflict of interest.

## Publisher's Note

All claims expressed in this article are solely those of the authors and do not necessarily represent those of their affiliated organizations, or those of the publisher, the editors and the reviewers. Any product that may be evaluated in this article, or claim that may be made by its manufacturer, is not guaranteed or endorsed by the publisher.

## Footnotes

^1^The variety of terms used to denote voluntary and involuntary attention may indicate that the distinction is not a strict dichotomy (Awh et al., 2012; Olk and Kingstone, 2015). This lack of a strict dichotomy between voluntary and involuntary attention is evidence for the possibility of a third kind of attention that is a recognizable mix between the two.

^2^In a recent literature review, Martella et al. (2020) conclude that the symptoms of ADHD are largely due to a dysfunction of the LC-NE system (Bertollo and Berchicci, 2014). The anterior insula plays an important role in the locus coeruleus-norepinephrine (LC-NE) system (Aston-Jones and Cohen, 2005; Clewett et al., 2014; Harsay et al., 2018; De Oliveira Sergio et al., 2021). According to van der Linden et al. (2021) the LC-NE system is a main factor in regulating activities characterized by intrinsic motivation.

^3^Casagrande et al. (2012) gesture in a similar direction when they say, “ADHD could depend on [subjects'] low level of arousal rather than being an independent disorder” (see also Bertollo and Berchicci, 2014). In a modified version of the ANT, Casagrande and colleagues had the flankers (animated fish) emit bubbles and a happy sound following correct responses, which, combined with an audio tone raising alertness, significantly improved performance in children diagnosed with ADHD. To us, this resembles a first step in the gamification of the ANT by making it more interesting for the subjects. The improved performance could very well be the result of an increase in intrinsic motivation, i.e., interest.
